# Fabrication of Gelatin/PCL Electrospun Fiber Mat with Bone Powder and the Study of Its Biocompatibility

**DOI:** 10.3390/jfb7010006

**Published:** 2016-03-04

**Authors:** Dongming Rong, Ping Chen, Yuchao Yang, Qingtao Li, Wenbing Wan, Xingxing Fang, Jie Zhang, Zhongyu Han, Jing Tian, Jun Ouyang

**Affiliations:** 1 Department of Orthopaedic, Zhujiang Hospital, Southern Medical University, No. 253, Gongye Avenue, Haizhu District, Guangzhou 510280, Guangdong, China; rong_dm1989@163.com (D.R.); cp19900717@163.com (P.C.); kamizj891026@126.com (J.Z.); hanzhongyu901001@aliyun.com (Z.H.); 2 Department of Anatomy, Guangdong Provincial Medical Biomechanical Key Laboratory, Southern Medical University, Baiyun District, Guangzhou 510515, Guangdong, China; gzyyc27@126.com (Y.Y.); mcqtli@scut.edu.cn (Q.L.); zwwb214@163.com (W.W.); Doc_Fang688@126.com (X.F.)

**Keywords:** gelatin, polycaprolactone, electrospinning, bone tissue engineering, scaffold

## Abstract

Fabricating ideal scaffolds for bone tissue engineering is a great challenge to researchers. To better mimic the mineral component and the microstructure of natural bone, several kinds of materials were adopted in our study, namely gelatin, polycaprolactone (PCL), nanohydroxyapatite (nHA), and bone powder. Three types of scaffolds were fabricated using electrospinning; gelatin/PCL, gelatin/PCL/nHA, and gelatin/PCL/bone powder. Scaffolds were examined using scanning electron microscopy (SEM) and transmission electron microscopy (TEM) observations. Then, Adipose-derived Stem Cells (ADSCs) were seeded on these scaffolds to study cell morphology, cell viability, and proliferation. Through this study, we found that nHA and bone powder can be successfully united in gelatin/PCL fibers. When compared with gelatin/PCL and gelatin/PCL/nHA, the gelatin/PCL/bone powder scaffolds could provide a better environment to increase ADSCs’ growth, adhesion, and proliferation. Thus, we think that gelatin/PCL/bone powder has good biocompatibility, and, when compared with nHA, bone powder may be more effective in bone tissue engineering due to the bioactive factors contained in it.

## 1. Introduction

The rapid development of biomedical engineering and modern surgery has greatly promoted the development of tissue engineering technology. More and more researchers put their attention on tissue engineering, especially on bone tissue engineering. Bone defects caused by trauma, infection, or tumor are always the focus of bone tissue engineering [1]. Bone transplantation is the main method to treat bone defects; bone transplantation includes autogenous bone transplantation, allogenic bone transplantation, and xenogeneic bone transplantation [2]. Currently, the gold standard of bone transplantation is still autogenous transplantation [3,4]. However, application of autogenous transplantation is restricted in many aspects, such as donor site injury and related complications and shortage of bone grafts; in addition, the harvested bone cannot be fabricated into the needed shape. Additionally, allogenic bone grafts and xenogeneic bone grafts have the advantages of rich sources and the convenience of harvesting. However, they can cause immunogenicity and infection, which can restrict their application [5,6,7,8]. All these problems compel researchers to find more ideal tissue substitutes. In 1977, Green *et al.* achieved chondroid tissue after they restored rabbit knee joint cartilage with rabbit chondrocyte-demineralized bone matrix composite [9]. Pereira *et al.* prepared scaffold of Mineralized poly(e-caprolactone)/gelatin core–shell nanofibers by co-axial electrospinning, and seeded human adipose-derived stem cells (hASCs) on the scaffold. They found that the scaffold can promote osteogenic differentiation of hASCs [10]. In addition, some researchers fabricated composite scaffolds with growth factors in them, and found that scaffolds with osteogenic growth factors can promote cell proliferation and differentiation. Li *et al.* fabricated a scaffold containing bone morphogenetic protein 2 (BMP-2) via electrospinning, and studied its biological properties with human bone marrow-derived mesenchymal stem cells (hMSCs); they found that a scaffold with co-processed BMP-2 supported a higher calcium deposition and enhanced transcript levels of bone-specific markers than in controls, thus, the scaffold can be a potential candidate for bone tissue engineering [11]. This provides an indication that, when we have proper scaffolds and cells, cells could differentiate into the new tissues or cells expected after cultivation under proper conditions.

Ideal scaffolds for bone tissue engineering should have the following properties: (i) biocompatibility; they should satisfy the common demands of medical materials, which are not toxic and do not degrade into toxic matters, without teratogenicity and tumorigenicity; (ii) biodegradability, in that they can degrade in accordance with tissue restoration, and that the biodegradability is tunable; (iii) good osteoconductivity, in that they can promote adhesion, growth, and related biological properties, and they are helpful for the import of oxygen and export of carbon dioxide, additionally, they can promote an ingrowth of vessels and nerves; (iv) plasticity, the materials can be fabricated into scaffolds of needed porosity and type, in addition, they should have considerable mechanical strength and fatigue resistance, so as to provide mechanical support; and (v) easy to obtain and at a low cost [12,13,14,15]. All in all, ideal scaffolds can create an environment in which cells can biologically adapt to them.

The objective of this study is to make a new biocompatible bone tissue engineering scaffold. We fabricate a gelatin/PCL electrospun fiber mat with natural bone powder; the scaffold, not only possesses the biocompatibility of gelatin and the mechanical strength of PCL, but also the rigidity of natural bone and all kinds of bioactive factors, which can promote the growth, proliferation, and differentiation of cells. All these advantages can make this scaffold an ideal candidate for bone tissue engineering.

## 2. Results and Discussion

### 2.1. Materials Characterization

#### 2.1.1. SEM Detection

**Figure 1:**
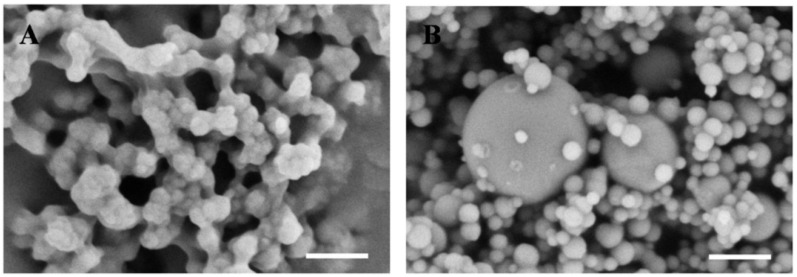
Scanning electron microscope images of bone powder (**A**) and nHA (**B**). Scale bar: 500 nm.

The bone powder used in this study was ground to micro-scale and nano-scale, as shown in Figure 1A. The larger particles may deposit during the electrospinning procedure, while the smaller parts can be united with gelatin/PCL fibers during the procedure. In addition, the diameter of nHA bought from Sigma (St. Louis, MO, USA) is smaller than 200 nm (Figure 1B), the larger parts in the picture are silica particles (acting as dopant), according to product instructions.

**Figure 2:**
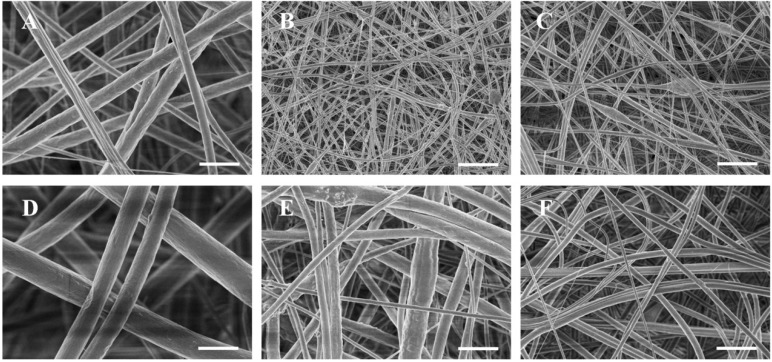
SEM images showing the morphology of fibers mat electrospun from gelatin/PCL solution (**A**), gelatin/PCL solution containing 10 wt % nHA (**B**), and gelatin/PCL solution containing 10 wt % bone powder (**C**). Images are shown at different magnifications to reveal surface structures ((**D**), (**E**) and (**F**) correspond to (**A**), (**B**) and (**C**), respectively). Scale bar: (A), (B) and (C), 10 μm; (D), (E) and (F), 2 μm.

Three types of scaffolds were successfully fabricated using the electrospinning method; gelatin/PCL fiber mat (Figure 2A), gelatin/PCL/nHA fiber mat (Figure 2B), and gelatin/PCL/bone powder fiber mat (Figure 2C). We can see from the SEM images that the fibers in scaffold gelatin/PCL are more smooth and the pores are larger; in addition, the porosity is higher than that of scaffolds gelatin/PCL/nHA and gelatin/PCL/bone powder. We can see that, when nHA and bone powder are electrospun into gelatin/PCL fibers, the fibers become rough, and the diameters tend to be smaller. nHA and bone powder can occasionally aggregate in some fibers to form bulges (Figure 2).

#### 2.1.2. TEM Detection

**Figure 3:**
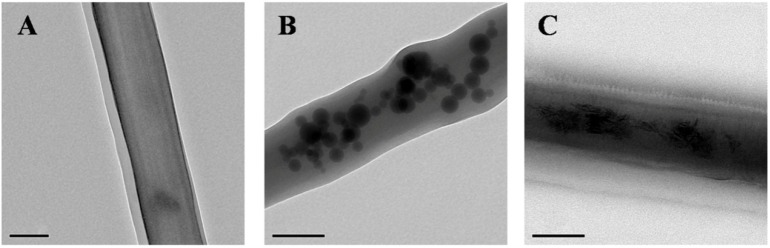
TEM images of gelatin/PCL (**A**); gelatin/PCL/nHA (**B**); and gelatin/PCL/bone powder (**C**). Scale bar: 200 nm.

We can conclude from the TEM-detection images that nHA and bone powder are successfully electrospun into the nanofibers of scaffolds gelatin/PCL/nHA and gelatin/PCL/bone powder, respectively, thus causing the positive effects of growth, adhesion, and proliferation of Adipose-derived Stem Cells (ADSCs) on scaffolds gelatin/PCL/nHA and gelatin/PCL/bone powder. Additionally, we can see from the TEM results that the nHA has a round shape, and bone powder has an irregular shape (Figure 3), while there are no particles in the fibers of scaffold gelatin/PCL, and the fibers are smooth and transparent (Figure 3).

#### 2.1.3. Diameter Distribution of Fibers in Each Group

**Figure 4:**
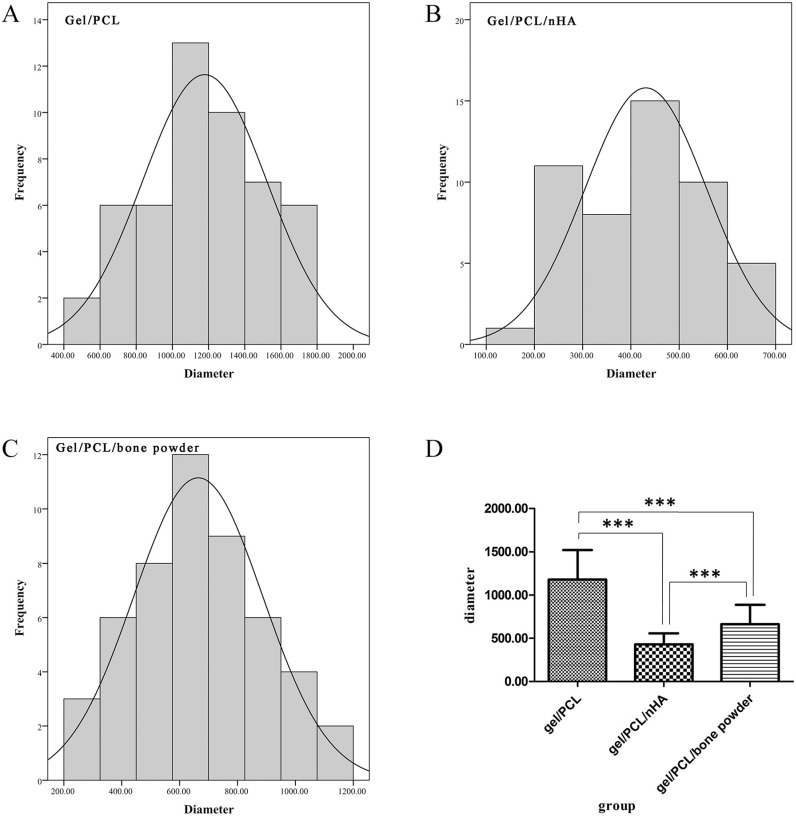
Diameter distribution and comparison of fibers in scaffold. (**A**) scaffold gelatin/PCL; (**B**) scaffold gelatin/PCL/nHA; (**C**) scaffold gelatin/PCL/bone powder; (**D**) diameter comparison of fibers in scaffolds gelatin/PCL, gelatin/PCL/nHA, and scaffold gelatin/PCL/bone powder. The asterisk (***) indicates statistically significant differences between the three groups (*P* < 0.001).

The diameter of fibers in scaffolds become smaller when nHA and bone powder are added, and the diameters of the fibers in scaffold gelatin/PCL/nHA are the smallest (Figure 4D). The diameters of nanofibers in the three groups are normally distributed (Figure 4A–C). Data was analyzed by SPSS 20.0 software and statistical differences were assessed using one-way analysis of variance (ANOVA), and there is a significant difference between the diameters of A, B, and C (*P* < 0.001), A > C > B.

### 2.2. Culture of ADSCs

**Figure 5:**
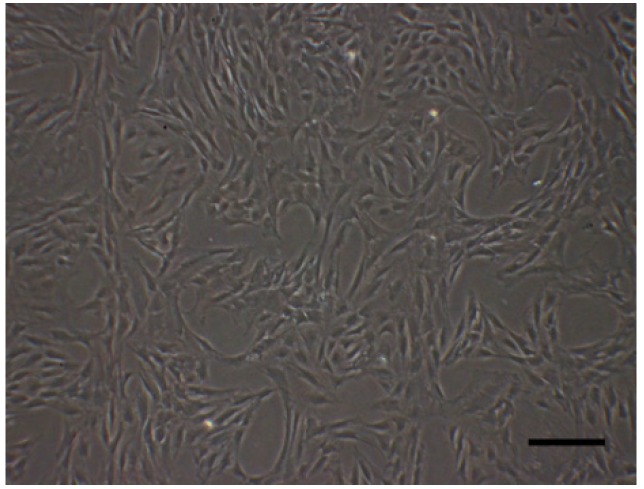
ADSCs of passage 3. Scale bar: 200 μm.

The third passage of ADSCs were in good condition (long, spindle shape and in group distribution) (Figure 5).

### 2.3. Cell Adhesion

**Figure 6:**
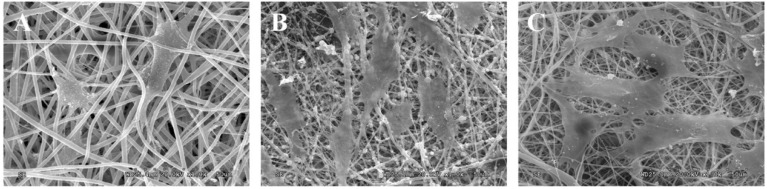
SEM images of ADSCs attached on scaffolds gelatin/PCL (**A**); gelatin/PCL/nHA (**B**); and gelatin/PCL/bone powder (**C**). Scale bar: 20 μm.

The SEM images revealed the morphologies of ADSCs attached to each scaffold. Cells covering the surface of the porous nanocomposites, and spreading with their pseudopodia, revealed better adhesion and activity (Figure 6). Additionally, we can see that the cells on scaffolds gelatin/PCL/nHA, and scaffold gelatin/PCL/bone powder are close to each other, thus we can conclude that biocompatibility of scaffolds gelatin/PCL/nHA and gelatin/PCL/bone powder are very good.

### 2.4. Cell Proliferation Assay

**Figure 7:**
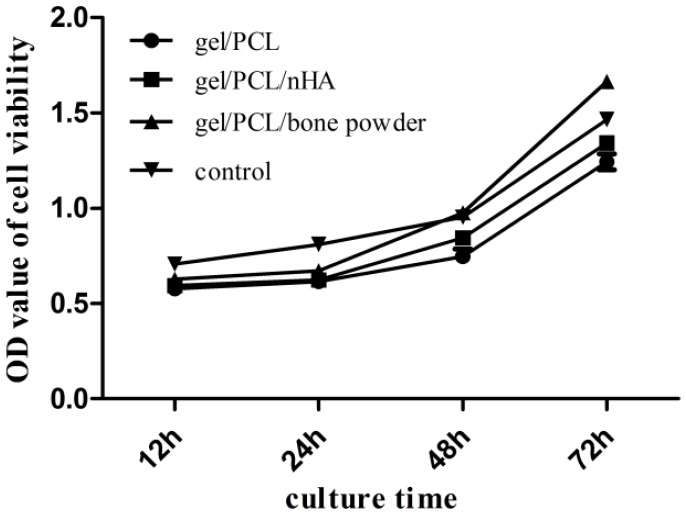
Comparison of cell proliferation between the three groups at time point 12 h, 24 h, 48 h, and 72 h (*n* = 3). OD, optical density.

With the CCK-8 assay, cell proliferation was measured at time points of 12 h, 24 h, 48 h, and 72 h, in each group. As is shown in Figure 7, there is no significant difference in proliferation rates between the cells on scaffolds gelatin/PCL, gelatin/PCL/nHA and gelatin/PCL/bone powder, and OD values are all lower than in the control group, possibly because of the negative effect of the scaffolds on cells; however, after 24 h, they proliferated to a high degree. In addition, cells on scaffold gelatin/PCL/bone powder proliferate faster than in the control group, at the time point of 48 h and the OD value is higher than in the control group. This positive effect may be caused by bioactive factors in scaffold gelatin/PCL/bone powder.

### 2.5. Cell Viability Analysis

**Figure 8:**
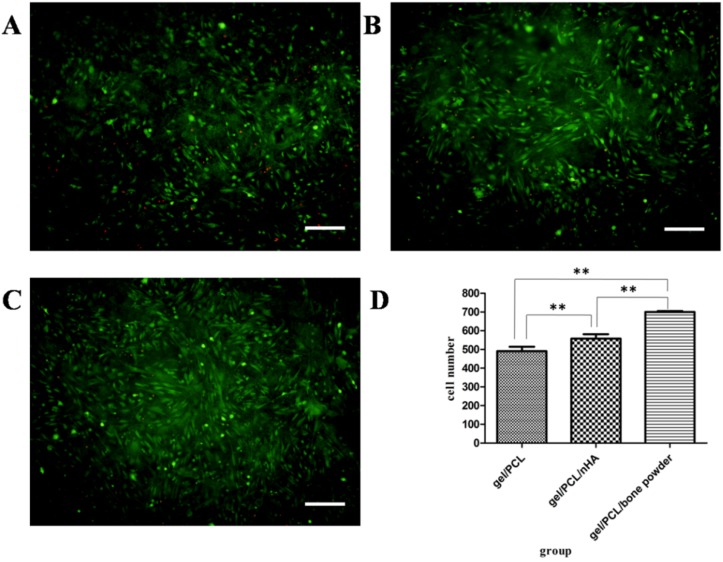
Viability analysis of ADSCs seeded on the three types of scaffolds after 48 h. (**A**) cells on gelatin/PCL fiber mat; (**B**) cells on gelatin/PCL/nHA fiber mat; (**C**) cells on gelatin/PCL/bone powder fiber mat; (**D**) counting of living cells on scaffold and comparison of each group. The asterisk (**) indicates statistically significant differences between the three groups (*P* < 0.05). Scale bar: 200 μm.

The cell viability of ADSCs in fiber mats were evaluated via Live/Dead staining at 2 days after cell seeding. Cells on fiber mats showed a homogeneous distribution. The presence of mainly green and few red cells indicated that cell viability was maintained on the fiber mats (Figure 8A–C). The number of living cells seems to be different in the three groups, and this is demonstrated by cell counting using Image Pro Plus 6.0 (Figure 8D).

### 2.6. Discussion

We fabricated a gelatin/PCL electrospun fiber mat with bone powder as a bone tissue engineering scaffold and studied its biocompatibility by seeding ADSCs.

In our study, we chose two Food and Drug Administration (FDA) approved, and widely used, materials as our basal components:PCL and gelatin. PCL is a biocompatible, biodegradable synthetic polymer with relatively high mechanical strength and low cost [16,17,18]. Moreover, the degradation rate of PCL *in vivo* is slow, and its by-products do not cause an acidic environment [19]. This is also an important reason why it is often chosen as a material for bone tissue engineering. When compared with natural polymers, the mechanical properties and biodegradability of synthetic polymers are tunable [20]. All these merits make PCL attractive in bone tissue engineering.

Obtaining polymer nanofibers using electrospinning technology is, more and more, attracting the attention of researchers, because the morphology of an electrospun nanofiber mat is similar to the protein fibers in a normal extracellular matrix. Though the electrospun PCL mats can mimic the structure of extracellular matrix (ECM) in living tissues, its poor hydrophilicity can cause a reduction in cell adhesion, migration, proliferation, and differentiation [21,22]. In order to improve the biomimetic property of PCL, we blend gelatin (a natural polymer) with PCL (due to its good biocompatibility and biodegradability). Moreover, when compared with collagen, the price of gelatin is lower [23]. Gelatin is a biocompatible and biodegradable natural polymer derived from collagen; it is an important part of the natural ECM, with no immunogenicity, but retains a RGD sequence to promote cell adhesion, proliferation, and differentiation [24,25]. Thus, gelatin can be electrospun with PCL to form composite scaffold with, not only good cell adhesion and proliferation properties, but also good mechanical strength. That is to say, the nanofiber mats fabricated using electrospinning can, not only mimic the structure of the ECM in living tissue, but also its chemical composition [26]. Recently, Gautam *et al.* indicated that a gelatin-modified PCL scaffold is beneficial to cell adhesion and proliferation as cell proliferation on a PCL–gelatin composite scaffold is faster than that of apure PCL scaffold [27].

In the preparation of biomimetic bone tissue engineering scaffolds, we should take the special microenvironment of the osteoblast into consideration, making scaffolds suitable for cell proliferation and osteogenic differentiation. Human bone is composed of organic and inorganic parts, and hydroxyapatite (HA) is the main component of the inorganic part, thus, in the preparation of bone tissue engineering scaffolds, hydroxyapatite has aroused a great deal of attention byresearchers covers proportion, morphology, and the size of HA.

HA has been widely used as all sorts of filling materials, and using it as a coating on the implant can increase the biological properties of the polymeric scaffold. Hydroxyapatite coating can, not only increase the biocompatibility of the implant, but also provide a bony surface for the implant, thus promoting bonding between implants and natural bone after implantation [28]. HA on the surface of the polymeric scaffold material can provide many benefits for scaffolds, such as increased bioactivity and osteoconductivity, and changing the composition and morphology of the scaffold [29,30]. It has been demonstrated that cell adhesion and proliferation are better on hydroxyapatite-modified scaffolds [31], and HA-modified scaffolds can promote the differentiation of mesenchymal stem cells into osteoblasts [32]. Budiraharjo *et al.* fabricated carboxymethyl chitosan scaffold with HA, and, by doing experiment, they come to the conclusion that osteoblasts on HA-coated scaffolds show better results with respect to adhesion, proliferation, and differentiation than those of scaffolds without HA [33].

The bone we used in this study is cortical bone from a femur. It can be processed into powders under low temperatures. SEM results (Figure 1A) indicated that the diameter of bone powders range from the micro-scale to the nano-scale, and it was demonstrated that the bone powders can be successfully electrospun into polymer fibers. The ingredients of the bone powder we used is in accordance with natural bone, that is to say:Bone powder not only contains inorganic parts, such as HA, but also organic parts, such as gelatin. Moreover, it contains all kinds of bioactive factors. Therefore, we assume that a gelatin/PCL/bone powder composite scaffold, not only has good biocompatibility, but also the good properties of promoting cell adhesion, proliferation, and osteogenic differentiation of ADSCs.

This is a pilot study of the fabrication and biocompatibility of scaffolds and, through this study, we can see that the scaffold containing a natural bone powder has good biocompatibility, and cells can adhere and proliferate well on this special scaffold. This will lay the foundation for latter study of its osteogenic conducting property *in vivo* and *in vitro*.

## 3. Experimental Section

### 3.1. Reagents and Devices

#### 3.1.1. Reagents

Trifluoroethanol (TFE), gelatin (gel), polycaprolactone and nano hydroxyapatite were all purchased from Sigma-Aldrich, Cortical bone from human corpse femur was abtained from Southern Medical University of donator. they were used to fabricate scaffolds in our study. Dulbecco’s Modified Eagle’s Medium (DMEM), fetal bovine serum, penicillin (100 units/mL), streptomycin (100 units/mL) were purchased from GIBCO BRL, they were used for culturing ADSCs. Live/dead staining kits was bought from Invitrogen Corporation to study cell vialibity. Cell counting kit-8 was abtained from Dojindo to study cell proliferation.

#### 3.1.2. Devices

Electronic balance was used to weigh the weight of materials, Electrostatic spinning equipment made by Beijing Ucalery Technology Development Co., Ltd. was used to fabricate scaffolds. NoVaTM Nano SEM 250 and FEI Tecnai G20 (FEI Company, Hillsboro, OR, USA) were used to characterized the scaffolds, inverted fluorescence microscope (Olympus BX51, Olympus, Tokyo, Japan) was used to take photos of cells, centrifugal machine (Sigma-Aldrich, St. Louis, MO, USA) was used to isolate ADSCs, cortical bone was processed by cryomill (Retsch, Haan, Germany), Clean Bench (Suzhou, China) is a clean workspace where cells were isolated. Cells were cultured in Hera 150 incubator (Thermo Scientific, Waltham, MA, USA), OD values were tested by Multiscan UV visible spectrophotometer (Tecan Schweiz AG, Männedorf, Switzerland).

### 3.2. Methods

#### 3.2.1. Materials Preparation

##### Preparation of Bone Powder

Firstly, cortical bone from a donated femur shaft was cut into small pieces, followed by immersion in ethyl alcohol absolute. After 24 h the pieces were dried at 4 °C. Then, the small bone pieces were ground into bone powder using acryomill.The powders were then sieved with a 200 mesh and washed with pure water and lyophilized to get rid of water. Finally, the bone powders were stored in liquid nitrogen for at least 3 months before use. The powders were ground in liquid nitrogen to anano-scale.

##### Preparation of Fiber Mats

Gelatin and PCL were, separately, dissolved in TFE under gentle stirring for at least 5 h to obtain a 10 wt % solution. Then we made a 1:1 mixture of the gelatin and PCL solutions. We prepared 3 types of solutions with the mixture: A is a solution of the mixture itself, B is a solution of the mixture with 10 wt % of nHA, and C is a solution of the mixture with 10 wt % bone powder. Consistent stirring and ultrasonic oscillation make nHA and bone powder spread evenly in the solutions. These solutions were individually loaded into plastic syringes (5 mL) and injected through a stainless steel, 18 gauge needle at a flow rate of 1.0 mL/h. The applied electric voltage was 17 kV and the distance between the spinneret and the collecting plate was 13cm. Under these conditions we fabricated three types of scaffolds: gelatin/PCL fiber mat, gelatin/PCL/nHA fiber mat, and gelatin/PCL/bone powder fiber mat. Before further experiments, they were separately punched into round pieces with diameters of 8 mm and 6 mm and sterilized using Co-60 radiation.

#### 3.2.2. Detection of Scaffolds

All the scaffolds were examined using scanning electron microscopy (SEM) and transmission electron microscopy (TEM).

#### 3.2.3. Isolation and Culture of ADSCs

Sprague–Dawley rats (3 weeks old) were anaesthetized using chloral hydrate, and then immersed in 75% ethanol for 5 min in order to sterilize them. Then, adipose tissue was obtained from inguinal fat pads. Isolation of ADSCs was accomplished, as described elsewhere [34]. Adipose tissue samples were first washed in cold PBS and then mechanically cut into mash. Later, 0.075% collagenase type I was added to the mash (about three times the volume of fat mash). Digestion was allowed for 40 min at 37 °C with gentle agitation. After centrifugation at 800 rpm for five minutes, we removed the floating adipocytes in the centrifugal tube and suspended the isolated cells in DMEM (containing 10% FBS and 1% penicillin⁄streptomycin). Then, cells were seeded on culture plates and put in an incubator at 37 °C and 5% carbon dioxide. Twenty-four hours later, culture medium was changed to eliminate non-adherent cells. Cells used in our experiments were passaged 3–5 times.

#### 3.2.4. Scanning Electron Microscopy Detection

After two days co-culture with the scaffolds, one piece of scaffold with cells on it was washed with PBS. Then, samples were fixed in 2.5% glutaraldehyde in PBS at 4 °C overnight, dehydrated using increasing concentrations of ethanol (50%, 70%, 80%, 90%, 95%, 99%, and 100%), and then treated with tertiary butanol. Finally, all the samples were lyophilized and sent for SEM detection.

#### 3.2.5. Cell Proliferation Assay

For the cell proliferation assay, the cells were seeded on scaffolds (6 mm) in 96-well plates. Cell proliferation was assayed by using Cell Counting kit-8, according to the manufacturer’s protocol for 12 h, 24 h, 48 h, and 72 h.

#### 3.2.6. Cell Viability Analysis

ADSCs were cultured on scaffolds in 24-well plate, and stained with live/dead staining kits. After co-culturing for 48 h, scaffolds with cells were washed using PBS and stained using a mixture of calcein AM (2 mM) and EthD-1 (4 mM) for 30 min in the absence of light, at room temperature. Samples were observed under fluorescence microscopy and images were randomly captured. The number of viable cells was counted by using Image-Pro Plus 6.0.

#### 3.2.7. Statistical Analysis

All assays were repeated with a minimum of *n* = 3 independent determinations for each data point, and data were presented as mean ± standard deviation (SD). Statistical data were analyzed using SPSS 20.0 software and statistical differences were assessed using one-way analysis of variance (ANOVA), followed by a *post hoc* Tukey’s Test. A value of *P* < 0.05 was considered as statistically significant.

## 4. Conclusions

We successfully fabricated three types of scaffolds; gelatin/PCL, gelatin/PCL/nHA, and gelatin/PCL/bone powder. We confirmed the characterization of the scaffolds using scanning electron microscopy (SEM) and transmission electron microscopy (TEM), and found that nHA and bone powders were successfully electrospun into fibers. Through cell cultures, we found that cell adhesion and proliferation are better on composite scaffolds with nHA and bone powder; in addition, the best results can be seen on scaffolds with bone powder. To sum up, nHA and bone powder can be electrospun into fibers to form gelatin/PCL/nHA and gelatin/PCL/bone powder composite scaffolds. Gelatin/PCL/nHA and gelatin/PCL/bone powder composite scaffolds showed better properties of cell adhesion and proliferation, and the gelatin/PCL/bone powder composite scaffold is the best candidate to mimic bone matrix and the microenvironment of osteoblasts. The fabrication of a gelatin/PCL/bone powder composite scaffold, and the study of its biocompatibility, can lay a foundation for later study of its osteogenic properties *in vivo* and *in vitro*. This project may provide new ideas for bone tissue engineering scaffold preparation; it is of great significance in further research of natural bone in clinical and fundamental orthopedics.
